# Improving the provision of Melbourne Health Familial Cancer services to Victoria’s Culturally and Linguistically Diverse (CALD) communities

**DOI:** 10.1186/1897-4287-10-S2-A56

**Published:** 2012-04-12

**Authors:** Y Bylstra, M Kentwell, GJ Lindeman, I Winship, L Hodgkin

**Affiliations:** 1 Familial Cancer Centre, Royal Melbourne Hospital, Victoria, Australia

## 

Heritable cancer syndromes affect all ethnicities, however, people from culturally and linguistically diverse (CALD) backgrounds do not attend the Royal Melbourne Hospital (RMH) Familial Cancer Centre (FCC) in the numbers that reflect the diversity of the Victorian population. Despite interpreters routinely organised, there still remains an impediment to attendance. Based on this observation, we considered the possible barriers preventing participation by CALD communities at the FCC, and developed initiatives to target these barriers.

Our first venture in trying to answer this question involved participation in the inaugural "Cancer Awareness Expo" in October 2010 organised by the Chinese Cancer Society of Victoria (CCSV). In preparation, we translated and printed the FCC brochure into traditional and simplified Chinese (suitable for Mandarin and Cantonese speakers, respectively) and these were available to the participants. Three RMH genetic counsellors were available throughout the day to engage with the participants with the assistance of interpreters. We learnt that, for this community, one of the main barriers to participation in the FCC is lack of awareness of our services.

We surmised that there would be other non-English speaking communities in Victoria for which language may limit awareness of our services. Therefore, we undertook to translate and print our FCC brochure into nine additional languages, namely Arabic, Croatian, Greek, Italian, Serbian, Somali, Spanish, Turkish and Vietnamese. We targeted these ten languages based on interpreter-use at the three hospitals the FCC services. We also established, using data collected by the Cancer Council Victoria, that people from these language backgrounds, are affected by breast and bowel cancer[1] – two of the cancer types that can be associated with an inherited predisposition.

As far as we are aware, this initiative, of producing an FCC brochure in languages other than English, is a first within Australia.

We have since taken further initiatives to raise awareness to CALD communities. We are involved with the “Living with Cancer” programs for Italian and Greek communities and are looking into establishing connections with other community groups. Our translated brochures have been sent to General Practitioners that speak one of these ten targeted languages and distributed amongst the oncology clinics within the hospitals that the RMH FCC services.

We believe that these ongoing initiatives are steps towards providing a more culturally-appropriate and equitable healthcare service to members of the CALD community in Victoria. We plan on continuing to engage with CALD communities, and evaluate the impact this has had on the number of patients who engage with the RMH FCC in the future.
